# Why do female sex workers disengage from targeted reproductive and sexual health services? Experiences from the Sisters with a Voice programme in Zimbabwe

**DOI:** 10.1186/s12913-025-12870-y

**Published:** 2025-07-03

**Authors:** Fortunate Machingura, Thomas Hartney, Galven Maringwa, Jasper Maguma, Sikhululiwe Mkwananzi, Rumbidzai Chipwere, Tariro Chinozvina, Primrose Matambanadzo, Gracious Madimutsa, Memory Makamba, Jeffrey Dirawo, M. Sanni Ali, Sarah Bourdin, Triantafyllos Pliakas, Amon Mpofu, Owen Mugurungi, Brian Rice, Andrew Phillips, James R. Hargreaves, Frances M. Cowan

**Affiliations:** 1 Centre for Sexual Health and HIV/AIDS Research Zimbabwe (CeSHHAR Zimbabwe), Harare, Zimbabwe; 2 Liverpool School of Tropical Medicine, Liverpool, UK; 3 London School of Hygiene and Tropical Medicine, London, UK; 4 National AIDS Council, Harare, Zimbabwe; 5 Ministry of Health and Child Care, Harare, Zimbabwe; 6 University of Sheffield, Sheffield, UK; 7 University College London, London, UK

**Keywords:** Preventive health services, Sex workers, Reproductive health services, Sexual health, Zimbabwe, HIV

## Abstract

**Background:**

The Sisters programme provides HIV and sexual and reproductive health services for female sex workers (FSW) in Zimbabwe. Many engage with these services only once, while others disengage after repeated visits. Little is known about reasons for disengagement and the extent of service needs after disengaging.

**Methods:**

Programme staff used site- and age-stratified random sampling to identify 1,200 programme records of FSWs who attended one of four Sisters clinics at least once between January 2018 and June 2019, and had no evidence of a further visit before September 2020. Outreach workers attempted to contact these FSWs via home visits, phone tracing and contacting peer educators. We calculated the proportion of FSWs successfully contacted, the level of ongoing engagement in sex work, expressed unmet need for Sisters services and the proportion of FSWs who subsequently made a return visit to the programme. We explored sociodemographic factors associated with these outcomes.

**Results:**

Of 1169 FSWs for whom contact was attempted, peer educators or others provided evidence in relation to 16 FSWs thought to have died. Of the 45% (504/1169) of FSWs who were successfully contacted, 37% (188/504) were no longer engaged in sex work, although 83% (156/188) reported that they were still in need of services. Reasons given for disengaging included having migrated (40%; 200/504); work commitments (16%; 79/504) and accessing services elsewhere (10%; 51/504). 62% of FSWs (313/504) said they were still active in sex work, among whom 23% (73/313) revisited the programme within 3 months of contact. FSWs living with HIV were less likely to re-engage with the programme (adjusted odds ratio 0.41, 95% CI 0.20–0.83). Age and site were associated with no longer being in sex work, while other factors showed no strong association.

**Conclusions:**

These findings highlight the need for robust outreach and re-engagement strategies that accommodate the mobility and evolving circumstances of FSWs. In particular, programmes that promote peer-led, community-based microplanning—supported by integrated data management systems—can help address stigma, frequent relocation, and financial constraints that hinder continuous care. By tailoring services to both active and former FSWs, health systems can ensure that essential sexual and reproductive health services remain accessible, even when FSWs exit sex work. Such differentiated approaches ultimately strengthen continuity of care, reduce service gaps, and support broader public health goals by improving health equity and outcomes for this high-risk population.

**Supplementary Information:**

The online version contains supplementary material available at 10.1186/s12913-025-12870-y.

## Background

Female sex workers (FSWs) globally constitute a small proportion of the population; however, they bear a disproportionate burden of HIV infection [1, 2]. The proportion of new infections estimated to be directly or indirectly attributable to commercial sex is high, and FSWs have up to 21 times higher risk of HIV acquisition than other women [3, 4]. FSWs represent a dynamic population with frequent internal and cross border migration [5]. Women’s engagement in sex work often fluctuates depending on diverse life course events and economic circumstances [6]. While effective tools for HIV prevention and treatment are available for this vulnerable group, their successful implementation necessitates unwavering commitment, consistent utilisation, and addressing persistent stigmatisation faced by individuals involved in selling sex [7, 8]. In sub-Saharan Africa (2023–2025), recent studies show a constellation of factors contributing to FSW disengagement, including entrenched stigma within healthcare settings, frequent relocation due to shifting economic opportunities, and fears of legal repercussions related to sex work criminalization [9, 10]. In Malawi, for example, mobile FSWs reported discontinuing HIV and sexual and reproductive health services after experiencing discriminatory treatment by clinic staff and harassment by local authorities [11, 12]. Such barriers compound existing financial constraints—such as travel costs and lost income during clinic visits—ultimately discouraging consistent service uptake [13–15]. Meanwhile, FSWs have also described a revolving door pattern of care engagement, characterised by repeated short-term exit-entry into services followed by renewed disengagement once stigma or economic stressors become overwhelming [16].

Understanding these disengagement drivers is not only crucial for FSW health but also for broader public health goals in high-burden regions. Because FSWs often serve as a “bridging” population—linking higher-risk sexual networks with the general population—unaddressed gaps in care can contribute to increased HIV transmission and unmet reproductive health needs [17, 18]. Moreover, the adverse social and economic outcomes associated with untreated infections and unintended pregnancies extend beyond individual FSWs to their families and communities [19, 20]. By identifying and mitigating these structural, social, and personal barriers, targeted programmes can bolster continuity of care, reduce the overall disease burden, and improve health equity among marginalised groups.

Peer-led interventions and microplanning have been shown to effectively enhance FSW engagement with HIV and sexual and reproductive health services [21]. These models rely on systematically strengthening outreach, often through peer educators who maintain “hotspot” lists, conduct rapid risk assessments, and tailor their support to individual needs [21]. By meeting FSWs where they are, microplanning can address common barriers—such as stigma, mobility, and inconsistent follow-up—while fostering trust and consistent service use.

The Sisters with a Voice programme (Sisters*)* provides comprehensive HIV and sexual and reproductive health services for FSWs across 86 clinics in Zimbabwe [22]. Beyond HIV care, services include family planning (oral contraceptives, injectables, implants, and intrauterine devices), pregnancy testing, antenatal care referrals, cervical cancer screening, and management of sexually transmitted infections. FSW diagnosed with HIV were until 2022, referred to government ART services; however, ART provision is now available through selected Sisters sites, including access to routine viral load monitoring. HIV prevalence among FSWs in Zimbabwe increases steeply with age, with an estimated HIV incidence of 3–6% per year [23]. Many FSWs who attend Sisters services engage regularly over several years, but nearly half do so only once [22].

Little is known about reasons for disengaging from targeted sexual and reproductive health programmes for FSW. Tracing studies in HIV treatment programme populations indicate that reasons for suspected loss to follow up can include inaccurate clinical records, relocation away from the treatment provider, undocumented transfer to another provider, disengagement from care, and death [24, 25]. Reasons for disengaging from care can also include improvements in health, difficulties with transport, religious beliefs, dissatisfaction with clinic services, competing demands from work and lack of money [25, 26]. Knowing why FSW who stop attending Sisters clinic services do so would help to understand whether they remain in need of HIV and sexual and reproductive health services.

The Sisters programme developed a pragmatic approach to use stored locator information (for which Programme attendees had given consent) to make contact with a sample of FSWs who had not returned for nine months, to support continuity of care. We report the steps involved in this approach, and outcomes of the process that attempted to locate and reengage FSWs. Among those who were contacted, we estimated unmet need for sexual and reproductive health services and tracked levels of re-engagement with services after contact had been made.

## Methods

### Study setting and population

Using clinical record and stored locator information, the Sisters programme attempted to identify and make contact with a location and age stratified sample of FSWs who had attended one of four purposively selected Sisters programme sites between 2009 and 2019 but had subsequently not visited the programme up to 30th September 2020. The programme operates in various locations including mobile clinics, highway clinics, and static clinics based in larger towns or cities. The programme purposively selected four sites based on operational readiness including the availability of experienced staff, consistent record-keeping, and established outreach systems to support tracing and re-engagement, making them suitable for piloting the approach prior to broader scale-up. Two sites (sites A and B) were small towns characterised by the presence of universities, mining activities, and commercial farming, with adjacent rural communal lands. Two were larger towns (sites C and D) connected to major highways that link Zimbabwe with neighbouring countries and were home to colleges, universities, and vibrant nightlife. The settings reflected varied urban/rural contexts and multiple sex work typologies, encompassing highway, bar, street, hotel, lodge, shebeen, and brothel-based sex work. Sisters’ clinics are located in sites with high rates of sex work and aim to cater to these varied typologies. By including different clinic types (mobile, highway, and static) and a variety of social and economic environments, the programme aimed to capture the breadth of experiences within the Sisters programme. Static clinic sites operate daily and have dedicated clinic and outreach staff stationed to provide services.

### Implementation

Although the Sisters programme was launched in 2009 and had expanded to 86 sites by 2020, this study focused on women who had accessed services during a more recent window. Specifically, we defined a sampling frame that included all FSWs who had attended one of the four selected Sisters clinics at least once between January 2018 and June 2019 and had no subsequent recorded visit before 30th September 2020. Clinical record and linked locator information was collected by the programme to facilitate contact with clients. We drew a site and age stratified random sample of 1,200 FSWs meeting these criteria, but with no unique identifier linked record of a further programme visit before September 2020. Relevant contact information routinely collected by the programme and kept securely (with written consent using a check box) is used by the programme for clinical follow up and includes names, alternative names, phone numbers, residential addresses, and a unique identifier code for identification of re-engagement with care at other Sisters clinics.

The programme team reviewed the extracted information to assess whether there was evidence of data errors or duplicate records. Subsequently, peer educators and program outreach workers at each site attempted to contact defaulting programme clients to assess their ongoing need for services, including whether they were still involved in sex work, had migrated, or relocated, were accessing services elsewhere, or died. Contacts were attempted by outreach workers in consultation with the local programme outreach team who maintain contact with FSWs in each site. The programme used locator information to attempt contact through home visits and phone calls. At each site, the outreach team made up to five home visit attempts unless a woman was confirmed to have relocated, died, or travelled. If no home address was available, or if a home visit was unsuccessful, the team attempted to contact the individual by phone. For each participant, the team made at least 10 and at most 15 phone calls on different days and at different times before concluding that someone was untraceable. This approach aligned with our tracing standard operating procedures, which specify that a case should be closed only if there is no reasonable chance of tracing the participant. These decisions were reviewed regularly by the tracing team, and if any new information emerged about a previously closed case, it was re-opened until the participant was located or definitively reclosed.

In March 2020, Zimbabwe introduced national lockdown measures in response to the COVID-19 pandemic. These included closures of public facilities, restrictions on gatherings, and stringent inter-district and inter-provincial travel restrictions, which were enforced intermittently through 2021. Although 10 static Sisters clinics reopened on 6 April 2020, and mobile services resumed on 18 May 2020, COVID-19-related restrictions affected the timing and implementation of the tracing activities. Home visits were delayed or rescheduled due to movement restrictions and roadblocks, while phone tracing was affected by limited or outdated contact information. Fieldwork was conducted in a staggered manner, depending on local operating conditions and in alignment with national COVID-19 protocols. The programme contacted participants from site A between 17th November and 8th December 2020, site B between 7th and 28th June 2021, site C between 3rd May and 2nd June 2021 and site D between 6th and 27th April 2021. These four clinics actively participated in the tracing exercise, during which, participants were asked about their current engagement in sex work and the reasons for disengagement with services. For FSWs still involved in sex work and in need of services, the programme facilitated their re-engagement and assisted in scheduling appointments if desired. The conversation guide for interviews was developed specifically for this study (see Supplementary file). Each step of the participant contact method was piloted among 134 female sex workers who had previously been classified as lost to follow-up. This group represented a convenience sample from one study site, comprising those whom the team was first able to attempt to re-contact during the pilot window. The pilot aimed to assess the feasibility and practicality of the re-engagement approach, ensuring the process was suitable for identifying eligible participants, confirming current engagement in sex work, and facilitating linkage back to services—prior to implementing the methods at scale across all selected sites. Comprehensive record-keeping practices were implemented at each stage of the tracing process to document outcomes described above. Although active tracing took place only at these four sites, we examined programme records across all 86 Sisters clinics to determine whether any of the traced FSWs had re-engaged with programme at one of the other programme sites, following contact by the tracing team, and to record any documented reasons for their re-engagement.

### Ethical considerations

This contacting exercise was conducted as an extension of existing programmatic data collection processes. The programme routinely asks all attendees if they will provide locator information, including names, addresses and phone numbers so that the programme can provide active monitoring of antiretroviral therapy (ART) or pre-exposure prophylaxis (PrEP). FSWs can opt to provide the information or decline to give it without affecting their access to care. The programme also records whether FSWs consent to being contacted by each method (phone, home visit) using a check box. If a woman indicated “*no contact”* for any method, no attempt was made to reach her through that channel. We used this locator information only for those FSWs who had previously agreed to be contacted. We did not re-seek consent since it had already been provided in the past. No data were collected from individuals who had refused contact in the programme’s records. Safety considerations were paramount and were taken into account by the program when determining the acceptability/feasibility of each contact attempt. Alternative methods of communication were explored if for any reason the outreach team felt that contacting a FSW directly might be unsafe. All outreach contacts were comprehensively recorded and aligned with the standard practices of the programme. Outcomes of the contact process were stored in the programme data systems. The intention to conduct this follow up exercise was outlined within the protocol for the AMETHIST group of studies, which received ethical approval from the Medical Research Council of Zimbabwe (MRCZ/A/2559), Liverpool School of Tropical Medicine (19-115RS) and the London School of Hygiene and Tropical Medicine (19123) [27]. The Medical Research Council of Zimbabwe reviewed and approved our application to conducted secondary statistical analysis of programme data (MRCZ/E/266). All data were de-identified prior to statistical analysis.

### Researcher positionality and reflexivity

Several of the authors are affiliated with the Centre for Sexual Health and HIV/AIDS Research Zimbabwe (CeSHHAR Zimbabwe), which implements the Sisters programme in partnership with the Ministry of Health and Child Care. The tracing and re-engagement processes were conducted as part of routine programme activities to support sustained engagement of key populations in care. This research draws on data generated through that operational process and was undertaken under the ethical frameworks outlined above.

We acknowledge that the intersection of implementation and research introduces important positionalities. The author team includes programme implementers, Zimbabwean public health researchers (from both CeSHHAR and the Ministry of Health), and UK-based academic collaborators. This heterogeneity enabled a combination of operational insight and methodological rigour, but also required careful reflexivity. Our proximity to the programme shaped both the framing and interpretation of the research. We approached this work with a reflexive stance, recognising how institutional affiliation, long-standing involvement in key population programming, and professional experience in a criminalised and stigmatised environment influenced our perspectives. To minimise bias and strengthen interpretive integrity, analysis was conducted using standardised procedures and supported by co-authors not directly involved in service delivery. These measures were intended to ensure critical distance while honouring the dignity and agency of a highly marginalised population.

### Statistical analysis

Statistical analyses were performed using Stata 17.0 (StataCorp LLC, College Station, TX, USA). We ran descriptive statistics to examine the socio-demographic characteristics (age, education, number of children and HIV status at registration) of sampled FSWs based on variables recorded at their first visit to the sister’s programme. A flow diagram was constructed to show the outcomes of the process: proportion successfully contacted, reasons for disengagement – including having ceased sex work, and number re-engaging with Sisters services within 3 months of contact.

Logistic regression analyses were used to investigate factors associated with no longer being in sex work among FSWs successfully contacted after missing clinic visits, and factors associated with re-engagement in care among those still engaged in sex work. Univariable logistic regression analyses were conducted for each explanatory variable, including site, age group, education level, marital status, number of children, and self-reported HIV status at registration. Multivariable logistic regression models were fitted using variables selected on the basis of theoretical relevance, rather than on the basis of statistical significance in the univariable analyses. Specifically, site, age group, education level, marital status and self-reported HIV status were included to account for potential confounding. The number of children was not included in the multivariable analyses due to a high proportion of missing data, which was correlated with age. Results were reported as odds ratios with 95% confidence intervals.

## Results

Of the 1,200 FSW sampled, 31 were not followed up further as 3 (< 1%) were identified as male partners of sex workers, 18 (2%) held no locator information and 10 (1%) reflected duplicate IDs. For the remaining 1,169 FSWs, their clinical records presented a name for 1,087 (93%), physical address for 765 (65%) and phone numbers for 891 (76%). Further investigation suggested 460/765 (60%) addresses reflected identifiable locations and 879 (99%) of recorded phone numbers were valid.

Median age of the 1,169 FSWs was 29 years (interquartile range 24 to 36). As shown in Table 1, and 199 (17.0%) were aged 16–19 years at the time of sampling. Of these, 58 (4.9%) were aged under 18 (16–17 years), while 141 (12.1%) were aged 18 or 19. The majority of FSWs had received secondary or higher levels of education (79.7%), and the largest group by marital status were FSWs who were separated, widowed, or divorced (70.5%). There was variation across sites for variables including education levels (see Table 1). Nearly all FSWs (94.6%) had at least one child at the time of registration with the Sisters programme, with nearly half (44.1%) having two or more children. On the basis of self-reported HIV status at first visit, 28.7% of those who reported their HIV status were positive, with higher rates observed among FSWs in Site B (40.8%) and lower in Site C (14.4%) (Table 1).

**Table 1:** Descriptive characteristics of sampled FSWs (*n* = 1,169)^†^

	Site A		Site B		Site C		Site D		Total	
	Freq.	%	Freq.	%	Freq.	%	Freq.	%	Freq.	%
Sampled client characteristics										
Total	115	100%	125	100%	463	100%	466	100%	**1169**	**100%**
Age in years (% total)										
16–19	7	6.1%	8	6.4%	92	19.9%	92	19.7%	**199**	**17.0%**
20+	108	93.9%	117	93.6%	371	80.1%	374	80.3%	**970**	**83.0%**
Median age (IQR)	31 (24–35)		33 (29–39)		27 (24-34.5)		30 (24–37)		**29 (24–36)**	
Education level										
None/primary	30	26.1%	49	39.2%	81	17.5%	59	12.7%	**219**	**18.7%**
Secondary and above	84	73.0%	69	55.2%	379	81.9%	400	85.8%	**932**	**79.7%**
Not reported	1	0.9%	7	5.6%	3	0.6%	7	1.5%	**18**	**1.5%**
Marital status										
Married/cohabitating	1	0.9%	0	0.0%	4	0.9%	15	3.2%	**20**	**1.7%**
Never married	31	27.0%	18	14.4%	103	22.2%	163	35.0%	**315**	**26.9%**
Separated/widowed/divorced	82	71.3%	107	85.6%	353	76.2%	282	60.5%	**824**	**70.5%**
Not reported	1	0.9%	0	0.0%	3	0.6%	6	1.3%	**10**	**0.9%**
Number of children										
None	6	5.2%	10	8.0%	26	5.6%	21	4.5%	**63**	**5.4%**
One child	39	33.9%	42	33.6%	143	30.9%	145	31.1%	**369**	**31.6%**
Two children	35	30.4%	33	26.4%	113	24.4%	99	21.2%	**280**	**24.0%**
Three children	26	22.6%	30	24.0%	98	21.2%	81	17.4%	**235**	**20.1%**
Not reported	9	7.8%	10	8.0%	83	17.9%	120	25.8%	**222**	**19.0%**
HIV status										
Negative	70	60.9%	68	54.4%	307	66.3%	267	57.3%	**712**	**60.9%**
Positive	39	33.9%	51	40.8%	129	27.9%	67	14.4%	**286**	**24.5%**
Not reported or unknown	6	5.2%	6	4.8%	27	5.8%	132	28.3%	**171**	**14.6%**

Note: Bolded values indicate statistical significance at *p* < 0.05

^†^Based on most recent programme data at point of sampling

**Fig. 1:**
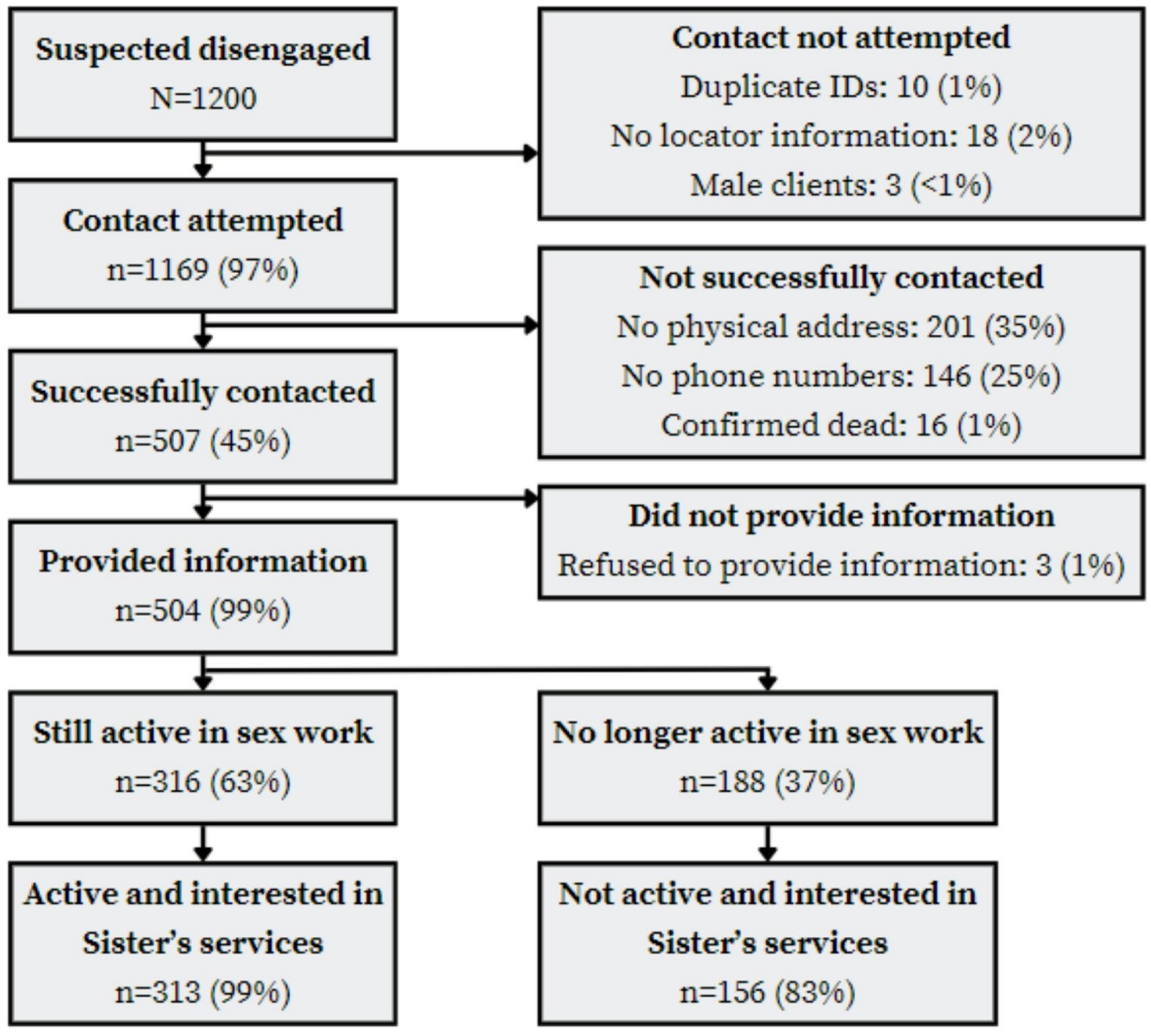
Participant flow diagram of contact outcomes

Contact with peer educators and/or those encountered during attempts to contact FSWs provided evidence that 16 FSWs had died (Fig. 1). Of 1169 FSWs, 43% were successfully contacted and provided feedback to the programme (504/1169). Of these, 313 (62%) reported that they were still actively involved in sex work and expressed interest in Sisters’ HIV, sexual and reproductive health services. The most commonly cited reason for disengagement from the Sisters programme was migration to another location within the same town or a different country (reported by 200 of 313 participants; 63.9%). This was followed by work commitments (79; 25.2%), accessing services elsewhere (51; 16.3%), and getting married (50; 16.0%). Less frequently mentioned reasons included issues with the service itself, such as a lack of necessary services (21; 6.74%), transportation costs (13; 4.2%), harassment at the clinic (10; 3.2%), and fear of exposure (5; 1.6%).

Among the 504 FSWs who were successfully contacted, 188 (37.3%) reported no longer being in sex work, of whom 156 (85%) also reported that they still had a need for Sisters services. Factors associated with no longer being in sex work (Table 2) included age, with those who were aged less than 25 or over 40 being more likely to no longer be in sex work. There was also a lower proportion of FSWs no longer in sex work among those with two or more children. In addition, there was variation across sites, with site B having a smaller proportion (10/60; 16.7%) no longer in sex work (adjusted odds ratio 0.29, 95% CI 0.12–0.68).

**Table 2:** Factors associated with no longer being in sex work (*n* = 188) among those interviewed (*N* = 504)

Explanatory variables	Number no longer in sex work / total interviewed (%)	Odds ratio (95% confidence interval)	*p*-value^*^	Adjusted odds ratio^**^ (95% confidence interval)	*p*-value
Site					
Site A	25 / 56 (44.6%)	**1.03 (0.57–1.85)**	**0.001**	**1.36 (0.72–2.59)**	**0.0045**
Site B	10 / 60 (16.7%)	**0.26 (0.12–0.53)**		**0.29 (0.12–0.68)**	
Site C	52 / 155 (33.5%)	**0.64 (0.42–0.98)**		**0.86 (0.54–1.37)**	
Site D (ref)	101 / 230 (43.9%)	**1.00**		**1.00**	
Age (years)					
16–19	35 / 62 (56.5%)	**4.07 (2.21–7.50)**	**< 0.005**	**2.85 (1.38–5.86)**	**0.005**
20–24	55 / 121 (45.5%)	**2.62 (1.59–4.31)**		**2.30 (1.33–3.98)**	
25–29	31 / 86 (36.0%)	**1.77 (1.01–3.10)**		**1.66 (0.92–3.00)**	
30–39 (ref)	42 / 174 (24.1%)	**1.00**		**1.00**	
40+	25 / 58 (43.1%)	**2.38 (1.27–4.45)**		**2.50 (1.31–4.77)**	
Education level					
None/primary	30 / 93 (32.3%)	0.76 (0.47–1.22)	0.251	1.05 (0.61–1.80)	0.8566
Secondary or above (ref)	155 / 401 (38.7%)	1.00		1.00	
Marital status					
Married/cohabitating	6 / 11 (54.5%)	**2.53 (0.76–8.48)**	**< 0.005**	2.10 (0.60–7.41)	0.2324
Never married.	64 / 126 (50.8%)	**2.18 (1.44–3.30)**		1.44 (0.87–2.38)	
Separated/widowed/ divorced (ref)	116 / 361 (32.1%)	**1.00**		1.00	
Number of children^***^					
None	8 / 18 (44.4%)	**0.94 (0.35–2.53)**	**0.002**		
One child (ref)	67 / 146 (45.9%)	**1.00**			
Two children	38 / 129 (29.5%)	**0.49 (0.30–0.81)**			
Three or more children	31 / 123 (25.2%)	**0.40 (0.24–0.67)**			
HIV status at registration					
Negative (ref)	105 / 286 (36.7%)	**1.00**	**0.007**	1.00	0.815
Positive	41 / 134 (30.6%)	**0.76 (0.49–1.18)**		0.96 (0.59–1.58)	
Not known	42 / 81 (51.9%)	**1.86 (1.13–3.05)**		1.19 (0.67–2.11)	

Note: Bolded values indicate statistical significance at *p* < 0.05

^*^Chi-squared test of independence

^**^Adjusted for site, age, education level, marital status and HIV status at original enrolment

^***^Excluded from multivariate model due to missing data (85 records), which was correlated with age. Number of records missing data for other variables were 2 missing age, 7 missing education and 3 missing marital status

Among the 313 FSWs who reported still being active in sex work and reported being in need of Sisters’ services, 72 (23.0%) re-engaged within three months (Table 3). This proportion was lower among those who were HIV positive at registration (16.1%), including in multivariate analysis, with those who were HIV positive at registration being less likely to re-engage with care (adjusted odds ratio 0.41, 95% CI 0.20–0.83). Amonog the 156 FSWs who were no longer active in sex work when interviewed but were interested in Sisters’ services, 16 (10.3%) re-engaged within three months.

**Table 3:** Factors associated with re-engagement into care (*n* = 124) among those still in sex work and in need of services (*N* = 313)^*^

Explanatory variables	Still in sex work and in need of services / re-engaged (%)	Odds ratio (95% confidence interval)	*p*-value^**^	Adjusted odds ratio^***^ (95% confidence interval)	*p*-value
Site					
Site A	6 / 31 (19.4%)	1.05 (0.39–2.84)	0.239	0.98 (0.34–2.81)	0.3616
Site B	16 / 50 (32.0%)	2.06 (0.98–4.32)		2.04 (0.88–4.74)	
Site C	26 / 103 (25.2%)	1.48 (0.79–2.77)		1.39 (0.69–2.80)	
Site D (ref)	24 / 129 (18.6%)	1.00		1.00	
Age (years)					
16–19	4 / 27 (14.8%)	0.59 (0.19–1.84)	0.23	0.67 (0.19–2.35)	0.5823
20–24	12 / 66 (18.2%)	0.76 (0.36–1.59)		0.81 (0.36–1.83)	
25–29	18 / 55 (32.7%)	1.65 (0.83–3.31)		1.53 (0.73–3.21)	
30–39 (ref)	30 / 132 (22.7%)	1.00		1.00	
40+	8 / 33 (24.2%)	1.09 (0.44–2.66)		1.22 (0.49–3.05)	
Education level					
None/primary	17 / 63 (27.0%)	1.31 (0.70–2.47)	0.397	1.17 (0.59–2.32)	0.6601
Secondary or above (ref)	54 / 246 (22.0%)	1.00		1.00	
Marital status					
Married/cohabitating	0 / 5 (0.0%)	0.00	0.144	0.00	0.2658
Never married.	10 / 62 (16.1%)	0.57 (0.27–1.18)		0.63 (0.28–1.44)	
Separated/widowed/ divorced (ref)	62 / 245 (25.3%)	1.00		1.00	
Number of children^****^					
None	2 / 10 (20.0%)	0.90 (0.18–4.58)	0.597		
One child (ref)	15 / 79 (19.0%)	0.84 (0.40–1.79)			
Two children	25 / 91 (27.5%)	1.36 (0.69–2.68)			
Three or more children (ref)	20 / 92 (21.7%)	1.00			
HIV status at registration					
Negative (ref)	50 / 181 (27.6%)	1.00	0.073	**1.00**	**0.0317**
Positive	15 / 93 (16.1%)	0.50 (0.27–0.96)		**0.41 (0.20–0.83)**	
Not known	7 / 39 (17.9%)	0.57 (0.24–1.38)		**1.08 (0.40–2.94)**	

Note: Bolded values indicate statistical significance at *p* < 0.05

^*^Excludes 16 individuals who re-engaged with services but were no longer in sex work when contacted

^**^Chi-squared test of independence

^***^Adjusted for site, age, education level, marital status and HIV status

^****^Excluded from multivariate model due to missing data (85 records), which was correlated with age. Number of records missing data for other variables were 2 missing age, 7 missing education and 3 missing marital status

## Discussion

In the context of a well-functioning, comprehensive sexual and reproductive health programme for FSWs in Zimbabwe, we attempted to make contact with 1169 FSWs who had been seen at least once from January 2018 to June 2019 but had no recorded subsequent visits by September 2020. We sought to understand their ongoing needs and contribute to programme improvement efforts. Among participants, 199 (17.0%) were aged 16–19 years, including 58 (5.0%) who were aged 16–17 at the time of sampling. We found that 35% FSWs had no recorded physical address and 25% no phone numbers in the system. Contact was successfully established with 504 individuals (43%), of whom 316 (63%) were still engaged in sex work and nearly all expressed ongoing interest in the services provided. Common reasons for disengagement from Sisters’ services were migration (63.9%), conflicting work commitments (25.2%), opting for alternative services (16.3%), and marriage (16.0%). Within three months of being traced 23.0% of the FSWs still in sex work re-engaged with services. Among 188 individuals who had ceased sex work activities, 83% also retained an interest in the services offered by Sisters, and 10.3% of these re-engaged.

This was our first systematic attempt to contact and re-engage FSWs in a comprehensive sexual and reproductive health programme using stored locator information. However, contacting efforts took place in-between lockdown periods during the COVID-19 pandemic, which caused severe disruption to both sex workers and the programme, making it more difficult for FSWs in need of Sisters services to attend clinics [28]. In addition, the dynamic nature of migration and evolving healthcare services means some FSWs initially perceived as disengaged may actually be receiving care elsewhere, potentially skewing our understanding of disengagement. Despite these challenges, our integrated approach—drawing on locator information, outreach workers, and cross-referencing with other sites—enhanced the reliability and validity of our findings. We identified factors that can inform future programme improvement and refinement, discussed further below. Overall, these data illustrate three ongoing challenges that need to be addressed by this programme and other targeted services [27].

The first challenge with contacting and re-engaging FSWs, is balancing an efficient, confidential service with efforts to ensure continuity of health support by collecting high quality locator information in the clinical record. Other programmes have also attempted to track vulnerable populations including people living with HIV (PLHIV) [29–31]. For instance, in Togo, 79.9% of PLHIV could not be reached using the phone numbers provided, with some lacking any phone contact information [29]. This can pose an obstacle to continuity of care in environments where home addresses may be dysfunctional or non-existent. In our setting with limited resources, phone calls are the primary method of contacting FSWs. Ensuring that phone contact information is collected for FSWs at their initial clinic visit and updated at each subsequent visit can support programme aims. A working phone number is one of the strongest predictors of successfully finding patients [30, 31]. Frequent home relocations, loss or change of phones or phone numbers, and damaged or changed contact details may contribute to the challenges of maintaining accurate and active phone records for FSWs. A study in Malawi revealed that poor contact information, update procedures and documentation were among the top reasons for explaining why patients may disengage from care [32]. Moreover, the failure to contact a significant number of individuals due to incorrect or missing locator information highlights the broader issue of data quality in health programs. This problem is not unique to the Sisters programme or this context, it has been documented in numerous health initiatives across sub-Saharan Africa, where inadequate infrastructure and record-keeping practices lead to incomplete or outdated patient information [33, 34]. The quality of collected data varies widely with data management being particularly challenging in many resource poor settings [35]. This can be due to poor infrastructure, a shortage of trained personnel, an unbalanced nurse-patient ratio, and a lack of investment by implementers and funders in electronic health records systems [36, 37]. Despite the Sisters programme’s investment in an electronic health records system, intermittent electricity, unreliable internet connectivity and, insufficient computer equipment often necessitate the continued use of a paper-based system, followed by delayed electronic updates. This dual process is both inefficient and error-prone. A robust, ‘electronic-first’ health management information system with automated prompts for regular contact information updates at each visit are in development.

Second, engagement in sex work is dynamic making it complex to track needs. In many cases we were unable to contact FSWs even where contact information were present. 37% of FSWs who were traced reported no longer being active in sex work. This suggests a possible hiatus or exit from sex work, or a change in circumstances that lessens their reliance on sex work while continuing to necessitate reproductive health service. The findings do however also indicate a persistent demand for services among active current and former FSWs, a population primarily within the reproductive age bracket. Notably, Sisters offers these services free of charge, whereas non-sex working FSWs are required to pay for them. This underscores the likelihood that this group of FSWs may continue to require sexual and reproductive health services, irrespective of their involvement in selling sex.

The inclusion of adolescents who self-identified as selling sex reflects the real-world population served by the Sisters programme, which adopts a harm reduction approach rather than excluding young people engaged in sex work. These participants received access to comprehensive sexual and reproductive health services—including HIV prevention, PrEP, and treatment—and were referred to social and protective services based on assessed needs and levels of vulnerability. Their presence in the study highlights the heightened health and social risks faced by minors involved in sex work, as well as the complex ethical and legal terrain of engaging this population in research and service delivery. It is also important to acknowledge that some of these adolescents may have first engaged with the programme at younger ages than recorded at the time of sampling, given the time lag between initial programme contact and data collection. In contexts marked by structural disadvantage, constrained agency, and early economic dependency, such realities demand enhanced safeguarding protocols, specialised support systems, and integrated, rights-based approaches that uphold adolescent protection while ensuring equitable access to care.

Third our investigations suggested that there may be room for improvement in retaining and re-engaging this population in support services. FSW may experience obstacles or hesitancy in seeking care. At the time of this work, Sisters with a Voice clinics were referring all HIV-positive FSWs to government antiretroviral therapy (ART) clinics, potentially diminishing the need for FSW-specific services. Since 2022, the Sisters programme has evolved to provide comprehensive care for ART patients. It now functions as a one-stop-shop where FSWs can access an extensive array of services, from healthcare to mental health and legal aid, thereby reducing the necessity for them to seek alternative services elsewhere. Nonetheless, the reduced re-engagement rate for HIV-positive FSWs underscores the importance of dedicated interventions and support to confirm their follow-up within government healthcare services. Furthermore, identifying and addressing structural, social, or healthcare barriers that impede their service utilisation and lead to their disengagement in care is crucial for creating targeted strategies to enhance the care engagement and retention of HIV-positive FSWs.

FSWs’ mobility and shifts in personal circumstances can lead to care discontinuation [38]. Studies from Uganda, South Africa, Zimbabwe, and Kenya found unsupportive family environments, healthcare discrimination against sex workers and people living with HIV, and inadequate quality of care as key factors in FSWs’ care disengagement [39–41]. These insights underscore the necessity for customised interventions catering to diverse groups, particularly active and HIV-positive FSWs. Additional factors such as inadequate services, transportation costs, clinic-related harassment, and fear of exposure were also cited, though for our programme we were pleased to see this was rare. We did not gather qualitative information as part of this study so were not able to explore the issue of harassment at clinics cited by a few FSWs further (it was only 10?). The programme staff were informed that this was raised as an issue. Ongoing training of clinic staff specifically addresses the need for all programme staff to treat attendees with dignity and respect. Further independent qualitative inquiry is required to better understand experiences of harassment and stigma among FSWs attending the programme. Evidence elsewhere highlights the role of transport costs, stigma, fear of disclosure, and competing life demands as common drivers of disengagement from care among vulnerable populations [25, 42]. This challenge is intensified for FSWs who may also be mothers and must accommodate their children during travel, increasing both transportation and childcare costs.

In settings with limited resources, financial limitations [43–45] and transport-related expenses [44, 46–48] are significant factors in patient attrition, especially when individuals face the tough choice of allocating scarce funds for transportation or essential needs like food for themselves and their families [49]. Negotiating transportation remains a challenge in resource-limited settings. Although arranging transportation for patients or reimbursing travel expenses [36, 50] may not be sustainable long-term solutions, alternative strategies must be explored. Regardless, ethical considerations concerning the provision of incentives must be carefully weighed, particularly in contexts of widespread poverty and limited resources. This study has identified reasons for disengagement, assisting Sisters with a Voice in customizing interventions to promote continuous care. Nonetheless, several limitations warrant consideration. Contacting efforts were undertaken between periods of COVID-19 lockdown, which may have affected both access to services and participant availability. Incomplete or outdated locator information for many FSWs hindered the ability to trace a large portion of those sampled, potentially biasing the findings toward individuals who were more stable or accessible. Additionally, for those who could not be reached, the reasons for disengagement remain unknown, which may limit the generalisability of results to the wider FSW population. While the Sisters programme provides a broad range of integrated HIV and SRH services to FSWs across Zimbabwe, there are structural limitations that affect the continuity and comprehensiveness of care. The programme’s reliance on self-reported data for service use and HIV status may introduce reporting bias. The high rate of one-time attendance presents persistent challenges for achieving sustained engagement and longitudinal follow-up.

## Conclusion

Overall, these findings emphasise the critical role that outreach and engagement strategies can play in maintaining the continuity of care for mobile and high-risk populations such as FSWs. Furthermore, the adoption of differentiated care models that include peer-led, community-based microplanning in tracing efforts could provide sustained, needs- and risk-specific interventions. The results also underscore the necessity for robust data collection and management systems to facilitate efficient patient tracking and service delivery. Enhancing the accuracy of locator data by linking visits across Sisters’ facilities can improve tracing and the targeting of interventions. Understanding the reasons for disengagement enables the Sisters programme to refine its tracing efforts in the future, optimising the outcomes of these initiatives. Incorporating a life course perspective in program design for FSWs, which caters to specific needs and challenges at various life stages, could enhance the continuity of care. Our focused examination of care re-engagement among FSWs is particularly significant for sustaining long-term access to HIV prevention services, contributing to wider public health objectives. Lastly, our emphasis on improved record-keeping practices promises more accurate outcome tracking and better-informed decision-making within the public health domain.

## Supplementary Information

Supplementary Material 1.

## Data Availability

Data can be made available upon request.

## Abbreviations

AMETHIST: Adapted Microplanning: Eliminating Transmissible HIV In Sex Transactions
ART: Antiretroviral therapy
CeSHHAR: Centre for Sexual Health and HIV AIDS Research Zimbabwe
COVID-19: Coronavirus disease 2019
FSW: Female sex workers
HIV: Human immunodeficiency virus
IQR: Interquartile range
MRCZ: Medical Research Council of Zimbabwe
PLHIV: People living with HIV
PrEP: Pre-exposure prophylaxis

## References

[1] Shannon K, Crago AL, Baral SD, et al. The global response and unmet actions for HIV and sex workers. Lancet. 2018;392(10148):698–710. 10.1016/S0140-6736(18)31439-9. 30037733 10.1016/S0140-6736(18)31439-9 PMC6384122

[2] UNAIDS. The path that ends AIDS: UNAIDS global AIDS update 2023. 2023. https://www.unaids.org/sites/default/files/media_asset/2023-unaids-global-aids-update_en.pdf. Accessed 11 Apr 2025.

[3] UNAIDS. Global HIV & AIDS statistics — 2022 fact sheet. 2022.

[4] UNAIDS. UNAIDS guidance note on HIV and sex work. 2012.

[5] Davey C, Cowan F, Hargreaves J. The effect of mobility on HIV-related healthcare access and use for female sex workers: a systematic review. Soc Sci Med. 2018;211:261–73. 10.1016/j.socscimed.2018.06.017. 29966821 10.1016/j.socscimed.2018.06.017

[6] Davey C, Dirawo J, Mushati P, Magutshwa S, Hargreaves JR, Cowan FM. Mobility and sex work: why, where, when? A typology of female-sex-worker mobility in Zimbabwe. Soc Sci Med. 2019;220:322–30. 10.1016/j.socscimed.2018.11.027. 30500610 10.1016/j.socscimed.2018.11.027

[7] Busza J, Matambanadzo P, Phiri L, Meki B. HIV prevention in individuals engaged in sex work. 2023:1–8. 10.1097/QCO.0000000000000891. 10.1097/QCO.0000000000000891 36729746

[8] Napierala S, Chabata ST, Fearon E et al. Engagement in HIV care among young female sex workers in Zimbabwe. J Acquir Immune Defic Syndr (1988). 2018;79(3):358–366. 10.1097/QAI.0000000000001815. 10.1097/QAI.0000000000001815 30036276

[9] Lichtwarck HO, Mbotwa CH, Kazaura MR, Moen K, Mmbaga EJ. Early disengagement from HIV pre-exposure prophylaxis services and associated factors among female sex workers in Dar Es Salaam, Tanzania: a socioecological approach. BMJ Glob Health. 2023;8(12). 10.1136/bmjgh-2023-013662. 10.1136/bmjgh-2023-013662 PMC10759139 38154811

[10] Eshikumo P, Awuor P, Blanco N, et al. Factors associated with retention in HIV prevention and treatment clinical services among female sex workers enrolled in a sex workers’ outreach program (SWOP) in Nairobi, Kenya. AIDS Behav. 2022;26(9):2969–80. 10.1007/s10461-022-03654-0. 35299260 10.1007/s10461-022-03654-0 PMC10256564

[11] Bossard C, Chihana M, Nicholas S, et al. HIV, sexual violence, and termination of pregnancy among adolescent and adult female sex workers in Malawi: a respondent-driven sampling study. PLoS ONE. 2022;17. 10.1371/journal.pone.0279692. 10.1371/journal.pone.0279692 PMC9803093 36584132

[12] Nantchito AV. Communication factors influencing antiretroviral therapy non-adherence among test and start clients in Zomba District, Malawi. MHBCC thesis. University of Malawi, The Polytechnic; 2021.

[13] Kabambe HLM. Exploration of female sex workers’ experiences and perceptions with accessing healthcare in Malawi: a qualitative study. PhD dissertation. University of Warwick; 2021. http://wrap.warwick.ac.uk/162176.

[14] Kumwenda MK, Mavhu W, Lora WS, et al. Feasibility and acceptability of a peer-led HIV self-testing model among female sex workers in Malawi: a qualitative study. BMJ Open. 2021;11(12). 10.1136/bmjopen-2021-049248.

[15] Lancaster KE, Powers KA, Lungu T, et al. The HIV care continuum among female sex workers: a key population in Lilongwe, Malawi. PLoS ONE. 2016;11(1). 10.1371/journal.pone.0147662. 10.1371/journal.pone.0147662 PMC4726447 26808043

[16] Dinse L. Barriers to exiting and factors contributing to the cycle of enter/exit/re-entering commercial sex work. PhD dissertation. Millersville University of Pennsylvania; 2018. https://millersville.tind.io/record/5007?v=pdf. Accessed 29 Mar 2025.

[17] Beattie TS, Adhiambo W, Kabuti R, et al. The epidemiology of HIV infection among female sex workers in Nairobi, Kenya: a structural determinants and life-course perspective. PLOS Global Public Health. 2024;4. 10.1371/journal.pgph.0001529. 10.1371/journal.pgph.0001529 PMC10773933 38190358

[18] Comins CA, Baral S, McIngana M, et al. ART coverage and viral suppression among female sex workers living with HIV in eThekwini, South Africa: baseline findings from the siyaphambili study. PLOS Global Public Health. 2024;4(5). 10.1371/journal.pgph.0002783. 10.1371/journal.pgph.0002783 PMC11111033 38776334

[19] Karlsen V. Kenyan NGOs’ fight towards a more inclusive society for female sex Workers in Mombasa. MSc thesis. University of Agder; 2024. https://uia.brage.unit.no/uia-xmlui/handle/11250/3156409. Accessed 29 Mar 2025.

[20] Elendu C, Amaechi DC, Elendu ID, et al. Global perspectives on the burden of sexually transmitted diseases: a narrative review. Med (United States). 2024;103(20):E38199. 10.1097/MD.0000000000038199. 10.1097/MD.0000000000038199 PMC11098264 38758874

[21] Cowan FM, Machingura F, Ali MS, et al. A risk-differentiated, community-led intervention to strengthen uptake and engagement with HIV prevention and care cascades among female sex workers in Zimbabwe (AMETHIST): a cluster randomised trial. Lancet Glob Health. 2024;12(9):e1424–35. 10.1016/S2214-109X(24)00235-3. 39151978 10.1016/S2214-109X(24)00235-3 PMC11345450

[22] Cowan FM, Chabata ST, Musemburi S, et al. Strengthening the scale-up and uptake of effective interventions for sex workers for population impact in Zimbabwe. J Int AIDS Soc. 2019;22(S4). 10.1002/jia2.25320. 10.1002/jia2.25320 PMC6643097 31328445

[23] Hargreaves JR, Mtetwa S, Davey C, et al. Cohort analysis of program data to estimate HIV incidence and uptake of HIV-related services among female sex workers in Zimbabwe, 2009–2014. J Acquir Immune Defic Syndr (1988). 2016;72(1):e1-e8. 10.1097/QAI.0000000000000920. 10.1097/QAI.0000000000000920 27093516

[24] Zürcher K, Mooser A, Anderegg N, et al. Outcomes of HIV-positive patients lost to follow-up in African treatment programmes. Trop Med Int Health. 2017;22(4):375–87. 10.1111/tmi.12843. 28102610 10.1111/tmi.12843 PMC5580236

[25] Rachlis B, Ochieng D, Geng E, et al. Evaluating outcomes of patients lost to follow-up in a large comprehensive care treatment program in Western Kenya. J Acquir Immune Defic Syndr (1988). 2015;68(4):e46–55. 10.1097/QAI.0000000000000492. 10.1097/QAI.0000000000000492 PMC4348019 25692336

[26] Alamo ST, Colebunders R, Ouma J, et al. Return to normal life after AIDS as a reason for lost to follow-up in a community-based antiretroviral treatment program. J Acquir Immune Defic Syndr (1988). 2012;60(2). 10.1097/FTD.0b013e3182526e6a. 10.1097/FTD.0b013e3182526e6a PMC3872063 22622076

[27] Cowan FM, Machingura F, Chabata ST, et al. Differentiated prevention and care to reduce the risk of HIV acquisition and transmission among female sex workers in Zimbabwe: study protocol for the ‘ AMETHIST ’ cluster randomised trial. 2022:1–12. 10.1186/s13063-022-06119-w PMC8917622 35279215

[28] Machingura F, Chabata ST, Busza J, et al. Potential reduction in female sex workers’ risk of contracting HIV during coronavirus disease 2019. Aids. 2021;35(11):1871–2. 10.1097/QAD.0000000000002943. 33973873 10.1097/QAD.0000000000002943 PMC8373442

[29] Saka B, Landoh DE, Patassi A, et al. Loss of HIV-infected patients on potent antiretroviral therapy programs in Togo: risk factors and the fate of these patients. Pan Afr Med J. 2013;15:1–7. 10.11604/pamj.2013.15.35.2198. 24009811 10.11604/pamj.2013.15.35.2198 PMC3758855

[30] Weigel R, Hochgesang M, Brinkhof MWG, et al. Outcomes and associated risk factors of patients traced after being lost to follow-up from antiretroviral treatment in Lilongwe, Malawi. BMC Infect Dis. 2011;11. 10.1186/1471-2334-11-31. 10.1186/1471-2334-11-31 PMC3039578 21272350

[31] Maskew M, MacPhail P, Menezes C, Rubel D. Lost to follow up: contributing factors and challenges in South African patients on antiretroviral therapy. South Afr Med J. 2007;97(9):853–7. 17985056

[32] Rachlis B, Ahmad F, Van Lettow M, Muula AS, Semba M, Cole DC. Using concept mapping to explore why patients become lost to follow up from an antiretroviral therapy program in the Zomba district of Malawi. BMC Health Serv Res. 2013;13(1):1. 10.1186/1472-6963-13-210. 23758879 10.1186/1472-6963-13-210 PMC3698212

[33] Yaya AJ, Asunmo AA, Abolarinwa ST, Onyenekwe NL. Challenges of record management in two health institutions in Lagos State, Nigeria. Int J Res Humanit Social Stud. 2015;2(12):1–9.

[34] Scorgie F, Chersich MF, Ntaganira I, Gerbase A, Lule F, Lo YR. Socio-demographic characteristics and behavioral risk factors of female sex workers in sub-Saharan Africa: a systematic review. AIDS Behav. 2012;16(4):920–33. 10.1007/s10461-011-9985-z. 21750918 10.1007/s10461-011-9985-z

[35] Mate KS, Bennett B, Mphatswe W, Barker P, Rollins N. Challenges for routine health system data management in a large public programme to prevent Mother-to-Child HIV transmission in South Africa. PLoS ONE. 2009;4(5):1–6. 10.1371/journal.pone.0005483. 10.1371/journal.pone.0005483 PMC2677154 19434234

[36] Makombe SD, Hochgesang M, Jahn A, et al. Assessing the quality of data aggregated by antiretroviral treatment clinics in Malawi. Bull World Health Organ. 2008;86(4):310–4. 10.2471/BLT.07.044685. 18438520 10.2471/BLT.07.044685 PMC2647428

[37] Forster M, Bailey C, Brinkhof MWG, et al. Electronic medical record systems, data quality and loss to follow-up: survey of antiretroviral therapy programmes in resource-limited settings. Bull World Health Organ. 2008;86(12):939–47. 10.2471/BLT.07.049908. 19142294 10.2471/BLT.07.049908 PMC2649575

[38] Karver TS, Barrington C, Donastorg Y, et al. Exploring the dynamics of the quality of HIV care experienced by female sex workers living in the Dominican Republic. PLOS Global Public Health. 2023;3(4):e0001479. 10.1371/journal.pgph.0001479. 37115734 10.1371/journal.pgph.0001479 PMC10146439

[39] Mtetwa S, Busza J, Chidiya S, Mungofa S, Cowan F. You are wasting our drugs: health service barriers to HIV treatment for sex workers in Zimbabwe. BMC Public Health. 2013;13(1):1–7. 23898942 10.1186/1471-2458-13-698 PMC3750331

[40] Duff P, Kipp W, Wild TC, Rubaale T, Okech-Ojony J. Barriers to accessing highly active antiretroviral therapy by HIV-positive women attending an antenatal clinic in a regional hospital in Western Uganda. J Int AIDS Soc. 2010;13:1–9. 20863399 10.1186/1758-2652-13-37 PMC2954932

[41] Scorgie F, Nakato D, Akoth DO, et al. I expect to be abused and I have fear: sex workers’ experiences of human rights violations and barriers to accessing healthcare in four African countries. Cape Town: African Sex Worker Alliance. 2011.

[42] Bothma R, Bello B, Scorgie F. Socio-demographic factors associated with retention of female sex workers in healthcare in hillbrow and Pretoria sex Worker Clinics. 2020.

[43] Dalal RP, MacPhail C, Mqhayi M, et al. Characteristics and outcomes of adult patients lost to follow-up at an antiretroviral treatment clinic in Johannesburg, South Africa. J Acquir Immune Defic Syndr (1988). 2008;47(1):101–107. 10.1097/qai.0b013e31815b833a. 10.1097/QAI.0b013e31815b833a 17971708

[44] Deribe K, Hailekiros F, Biadgilign S, Amberbir A, Beyene BK. Defaulters from antiretroviral treatment in Jimma university specialized hospital, Southwest Ethiopia. Trop Med Int Health. 2008;13(3):328–33. 10.1111/j.1365-3156.2008.02006.x. 18298607 10.1111/j.1365-3156.2008.02006.x

[45] Dahab M, Charalambous S, Hamilton R, et al. That is why I stopped the ART: patients’ & providers’ perspectives on barriers to and enablers of HIV treatment adherence in a South African workplace programme. BMC Public Health. 2008;8:1–6. 10.1186/1471-2458-8-63. 18282286 10.1186/1471-2458-8-63 PMC2276211

[46] McGuire M, Munyenyembe T, Szumilin E, et al. Vital status of pre-ART and ART patients defaulting from care in rural Malawi. Trop Med Int Health. 2010;15(SUPPL 1):55–62. 10.1111/j.1365-3156.2010.02504.x. 20586961 10.1111/j.1365-3156.2010.02504.x

[47] Palombi L, Marazzi MC, Guidotti G, et al. Incidence and predictors of death, retention, and switch to second-line regimens in antiretroviral- treated patients in sub-Saharan African sites with comprehensive monitoring availability. Clin Infect Dis. 2009;48(1):115–22. 10.1086/593312. 20380075 10.1086/593312

[48] Brinkhof MWG, Dabis F, Myer L, et al. Early loss of HIV-infected patients on potent antiretroviral therapy programmes in lower-income countries. Bull World Health Organ. 2008;86(7):559–67. 10.2471/blt.07.044248. 18670668 10.2471/BLT.07.044248 PMC2647487

[49] Tuller DM, Bangsberg DR, Senkungu J, Ware NC, Emenyonu N, Weiser SD. Transportation costs impede sustained adherence and access to HAART in a clinic population in Southwestern Uganda: a qualitative study. AIDS Behav. 2010;14(4):778–84. 10.1007/s10461-009-9533-2. 19283464 10.1007/s10461-009-9533-2 PMC2888948

[50] Miller CM, Ketlhapile M, Rybasack-Smith H, Rosen S. Why are antiretroviral treatment patients lost to follow-up? A qualitative study from South Africa. Trop Med Int Health. 2010;15(Suppl 1):48–54. 10.1111/j.1365-3156.2010.02514.x. 20586960 10.1111/j.1365-3156.2010.02514.x PMC3060335

