# Supplementary material for: Why do female sex workers disengage from targeted reproductive and sexual health services? Experiences from the Sisters with a Voice programme in Zimbabwe
Source: BMC Health Serv Res. 2025 Jul 3;25:915. doi: 10.1186/s12913-025-12870-y (PMC12225132; doi:10.1186/s12913-025-12870-y)
Supplement: Supplementary file 1 — Supplementary Material 1. [file 12913_2025_12870_MOESM1_ESM.pdf]

12.2 Conversation guide [1]

After the verification process. Introduce yourself as follows:

My name is …... I am visiting (*or phoning if visits fail)* you from the X Sisters clinic. Is this a good time for us to talk, maybe for about 8 minutes? *[Assuming yes],* hey as our records suggest you haven't returned to any Sisters clinic for more than 6 months... You know that we should attend the programme every 3 months. We offer a range of services, including some that were not available when you visited previously. Are you still in need of Sister's services at X Clinic?
[If yes] – Should I make an appointment for you to come on [Date]? Or would you like me to take you to the clinic today if you want?
[If no] – ask why? Show interest and nod while engaging in their talk…Ahaa…, I understand…, is that why have you not revisited FSW programme since the last visit? [they state the reasons or reaffirm]. Would that be all? Are there other reasons that have made you delay/stop your visits to the clinic?
Are you still selling sex? [*if not when did you stop? Why did you stop?]* Are you visiting any health services other than sisters? When was last time you went there? Oh, I see…, okay okay…., thank you for sharing this information with me…. are there other services that you need that are not offered by the sisters' clinic? [probe what these are and why they are needed]. Ah…, yaaa…, okay, okay. that's very important. *[Where they mention services that you know are provided at the clinic, but they think are not offered, correct immediately and refer the appointment again]*…ah, I'm sorry when you went the last time you did not get this/these service(s), but actually, the clinic provides this/these. If you like I can give you an appointment and the clinic staff will make sure that you get this/these. If you face any problems, do not hesitate to call me if you like. Can I give you my number? You can call me anytime if you want? [If the services are genuinely not provided, agree with them]. Ah, yeah that's an essential service, and it's unfortunate it is not offered at the clinic, do you now get this elsewhere?" where?

Thanks a lot for sharing this…this is a constructive discussion. Hey, I have some condoms with me, do you want some? I don't want to take much of your time *[mention their name],* are you okay with me following up on you again next time if you don't return to the clinic, to check on you? If so, how best can we contact/find you? *[Probe for information to update the locator form (Annex 9.4), e.g. phone number, address etc.]* . Ah very well, thank you so much and stay in touch.

Record the responses in the Tracing assessment form attached in Annex 9.3
